# Exploring the barriers to the antiretroviral therapy adherence among people living with HIV in Bangladesh: A qualitative approach

**DOI:** 10.1371/journal.pone.0276575

**Published:** 2022-10-21

**Authors:** Fariha Hossain, Mahmudul Hasan, Nilufar Begum, Devi Mohan, Sharuna Verghis, Nowrozy Kamar Jahan

**Affiliations:** 1 Jeffrey Cheah School of Medicine and Health Sciences, Monash University Malaysia, Selangor Darul Ehsan, Malaysia; 2 Ashar Alo Society (AAS), Dhaka, Bangladesh; FHI360, UNITED STATES

## Abstract

**Introduction:**

Since the evolution of highly active antiretroviral therapy (ART), a near-perfect ART adherence level (>95%) is needed to control viral suppression. Non-adherence to treatment may lead to acquired immunodeficiency syndrome (AIDS) and drug resistance. Though the Bangladesh government provides free treatment and counselling services to people living with human immunodeficiency virus (PLHIV), only 22% of the identified PLHIV continue treatment. Therefore, this study aims to explore the barriers that obstruct the Bangladeshi PLHIV to ensure ART adherence.

**Methods:**

We conducted a qualitative study in Dhaka, Bangladesh, and recruited the sensitive study population following non-probability, mainly purposive sampling from a community-based registered organization for PLHIV. We conducted the in-depth interview using a semi-structured guideline with 15 consented respondents. We transcribed the audio-recorded interviews in the local language (Bangla) and then translated those into English for data analysis. During the data extraction process, the lead and corresponding authors independently extracted raw data to generate different themes and sub-themes and invited other authors to contribute when they could not solve any discrepancies.

**Results:**

The study identified three significant categories of barriers at the individual, community, and institutional levels that negatively interfered with ART adherence. The most dominant barriers were discrimination and rejection related to stigma, as almost all participants mentioned these barriers. Stigmatizing attitudes and the discriminatory act of the community people and healthcare providers critically affected their treatment adherence. Other leading barriers were improper inventory management of ART-related medicines and CD4 tests and lack of proper counselling. In addition, we found that a positive approach toward life and family support motivated some respondents to overcome the barriers.

**Conclusions:**

We recommended strengthening Bangladesh’s HIV/AIDS prevention, treatment, and management program with a special focus on the improvement of the supply chain of ART-related medicines and CD4 tests and ensuring proper counselling. In addition, we recommended strengthening the behaviour change communication and IEC activities at a large scale to destigmatize health facilities and community levels.

## Introduction

Antiretroviral therapy (ART) is a recommended lifelong treatment for those who are living with human immunodeficiency virus (HIV), and non-adherence to ART may lead to disease progression, which may cause acquired immunodeficiency syndrome (AIDS) and drug resistance [1]. Since the evolution of highly active ART, medication adherence has become the most crucial issue in HIV treatment and management [2, 3]. Studies demonstrated that viral load differs with the different levels of treatment adherence [4–6]. To achieve sustainable viral suppression, a near-perfect ART adherence level (>95%) is needed [7, 8], and even if the treatment adherence level is between 90%-95% instead of >95%, it shows a significant decrease in viral suppression [9–11].

Though the first HIV case was detected in Bangladesh in 1989 [12], the national prevalence among the general population is relatively low (less than 1%) [13]. HIV prevalence is mainly concentrated in high-risk behaviour groups like commercial sex workers, male intravenous drug users, transgender, professional blood donors, migrant workers, and long-distance truck drivers [12, 14–17]. UNAIDS estimated that until 2018, 14,000 people might be living with HIV in Bangladesh; among them, only 5100 (36%) were officially diagnosed as people living with HIV (PLHIV). Though the Bangladesh government provides free treatment and counselling services to PLHIV with the support of UNAIDS, WHO, UNICEF, and UNFPA, only 3045 PLHIV were enrolled in the ART, i.e., 22% ART coverage [18, 19]. This information indicates that there are some barriers to getting access to free treatment and ensuring its adherence, which is hindering the success of the Bangladeshi government’s support for HIV-related treatment programs. In addition, new HIV infection is maintaining an increasing trend in Bangladesh; there were an estimated 1000, 1400, and 1600 new infection cases in 2010, 2015, and 2018, respectively [18]. These new PLHIV may hesitate to start the ART treatment if the obstacles remain.

To our knowledge, no study has been conducted in Bangladesh to generate evidence on the barriers to ART adherence from PLHIV’ perspective. This qualitative study aims to explore the barriers that obstruct the Bangladeshi PLHIV from starting the treatment and ensuring ART adherence. The study intends to provide evidence to Bangladesh’s health policymakers to strengthen the implementation of effective policy and appropriate interventions to overcome the obstacles to increasing ART adherence; hence, it will contribute to increasing ART coverage.

## Materials and methods

### Ethical statement

We received the ethical clearance from the Monash University Human Research Ethics Committee (MUHREC) on March 22, 2019, and the Ethics ID number is 17777. This study strictly adhered to the ethical guidelines set by Monash University. Accordingly, we informed the study respondents about their voluntary participation through the translated explanatory statement in the local language (Bangla). We also informed them that they could withdraw themselves from the study at any time, refuse to answer any questions if they want, and have no or minimal risk if they join the study because all the information will be kept confidential. They were de-identified with a unique identification number upon enrolling in the study.

### Study design

To achieve the research objective, we conducted a qualitative study to explore and collect the experiences of PLHIV living in the community regarding the obstacles to starting and continuing their ART. We collected the data through an in-depth interview, an essential qualitative research technique, which allowed us to explore the respondents’ experiences regarding the barriers to their ART adherence and how they could overcome some of the barriers, if any.

### Study site

We conducted the study in the Ashar Alo Society (AAS), a community-based registered non-profit organization mainly working to provide support, care, and treatment to PLHIV in Bangladesh. AAS started to work as a self-help group for PLHIV in 1998. Since then, it has worked as a PLHIV network office for all PLHIVs living in Bangladesh. It is one of the leading treatment centres where PLHIV receives free of charge ART, sponsored by the Bangladesh government.

We selected this organization as a study site because it is challenging to identify the sensitive study population like PLHIV in the community due to stigma and fear of disclosure. On the other hand, AAS had 2828 registered members of PLHIV till December 31, 2018. Specifically, at the Dhaka centre where we conducted this study, the total number of registered PLHIV members was 1479; out of them, 924 were male, 478 were female, 57 were children, and 20 were transgender. PLHIV members often visit AAS to meet their peers and receive different kinds of training and treatments. That is why the research team selected the AAS premises as a study site to recruit sensitive study respondents.

Before conducting the study, we received formal approval from the Executive Director of AAS, introducing the research team to the senior officials and senior counsellors. This formal approval and introduction facilitated the research team to start the data collection process with the sensitive PLHIV respondents. AAS had trained senior counsellors to provide counselling services to PLHIV and their family members. We collected data in a private room next to these senior counsellors’ rooms to ensure the safety of both researchers and respondents. It also created a comfortable interview environment for study respondents ensuring their safety and privacy. Senior counsellors also made a counselling service available for the study respondents if they needed it, especially if they felt any distress while giving information on their illness and barriers to treatment adherence.

### Study population and recruitment process

We recruited the study respondents from the registered members of the Dhaka centre of AAS. Our selection criteria were that the study respondents should be adults, aged 20 years and above, either male or female or transgender, and at least receiving the ART for one year or more. We selected the sample using non-probability, mainly purposive sampling. As the research team could get access to recruit the sensitive study respondents on the AAS premises, we can also consider it a convenience sampling. Besides, it was a heterogeneous sampling, as the samples have a wide range of variation regarding their socio-demographic characteristics, which is presented in Table 1 of the result section.

**Table 1:** Summary profile of the study respondents.

Different categories	Sub-categories	Number
Age		
Mean	42	
Minimum- Maximum	25 and 55	
Gender		
	Male	11
	Female	4
Education status		
	Illiterate	1
	Primary	4
	Secondary incomplete	6
	Secondary complete	3
	Masters	1
Employment status		
	Unemployment	3
	Agriculture	1
	Service holder	4
	Business/small business	4
	Laundry worker/Tailor	2
	Housewife	1
Year of diagnosis		
	2004	1
	2005	2
	2006	2
	2009	3
	2010	3
	2011	2
	2012	2
Starting year of ART		
	2005	3
	2007	2
	2009	2
	2010	2
	2011	2
	2012	3
	2014	1

To avoid selection bias, we invited the potential study respondents to join the study through an advertisement in the local language Bangla, which was openly displayed on AAS’s notice board as a poster. This approach was adopted to provide equal opportunity to the sensitive study population like PLHIV. Later, we distributed the Bangla-translated explanatory statement among those potential study respondents who expressed interest in joining the study after knowing about it from the advertisement.

Our Bangladeshi research assistant (RA), who had extensive experience conducting qualitative research in the HIV/AIDS field, was available physically at the AAS premises during office hours (9 am to 5 pm) and also through mobile phone during the weekend to answer any queries if the potential respondents had. The RA was also available, physically or over the phone, to provide additional information about the study, primarily how the study would be conducted if the potential respondents wanted to know. Thus, we allowed the potential respondents to think carefully before joining the study. Finally, we recruited 15 PLHIV as study respondents.

### Data collection

Before collecting data, we collected written consent in Bangla from the 15 consented study respondents. The research team also received consent for the audio recording of the interview to capture the detailed information accurately. At the beginning of the interview, we asked a few close-ended questions to collect their background information and medical history. Then, we conducted the in-depth interview in the local language, Bangla using a semi-structured interview guideline (S1 Table).

Open-ended questions allowed us to collect the data to assess their treatment adherence and explore their experiences regarding any barriers they faced throughout the treatment period and how they overcame all or some of those barriers. The interview took approximately 30–40 minutes per respondent. Though the data was saturated at the 9th interview, we continued the data collection until the last (15^th^) respondents who wanted to provide their information, and we also did not want to disappoint them by not collecting their data.

### Data analysis

The audio-taped interviews were transcribed in Bangla (the local language) and then translated into English for data analysis. Before analysis, the authors checked the transcribed and translated data collected by the research assistant. Therefore, they imported the raw data into NVivo software for data extraction. Two authors (the lead and corresponding authors) independently extracted data and generated different themes and sub-themes during the data extraction process. They invited the other authors to contribute when they could not solve any discrepancies. While doing data analysis and thematically categorizing the study findings, authors gave emphasis on the participants’ narration and repetition according to the thematic analysis guidelines [20, 21].

## Result

Fifteen consented respondents (11 were male, and four were female), who met the selection criteria, joined the study. The summary profile of the respondents, including their socio-demographic characteristics and medical history, are summarized in Table 1.

The medical history consisted of the year when HIV was diagnosed and when they started their ART. We also collected data on their treatment adherence status and found that at the time of data collection, all were continuing the treatment, but they faced different kinds of barriers to continuing their treatment.

**Fig 1:**
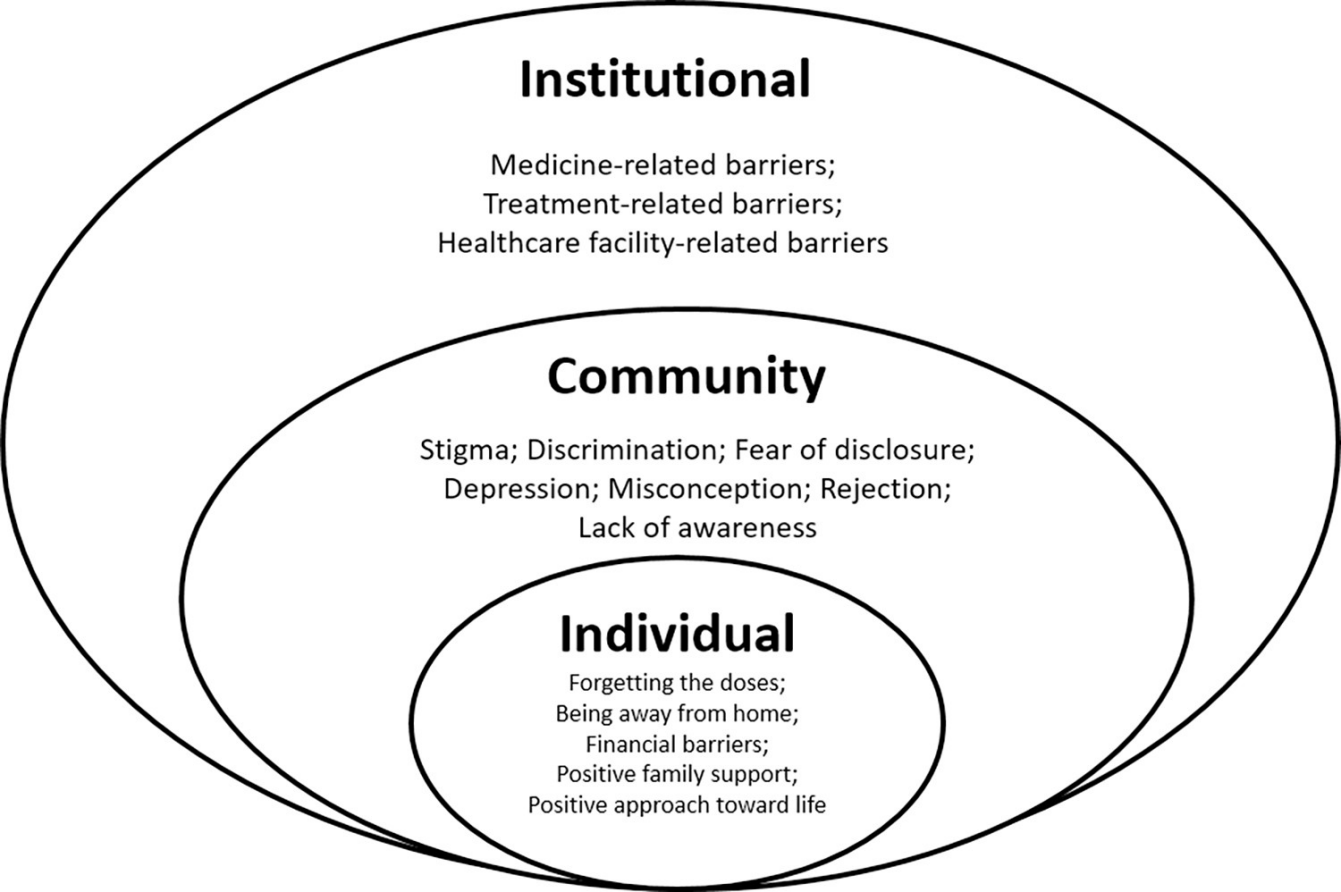
Thematic framework according to the socio-ecological model.

The respondents’ ages ranged from 25 to 55 years; four respondents completed secondary education and above. At the time of data collection, 11 respondents were employed in different sectors, starting from agriculture to business; four respondents still could continue their employment in an office environment after being PLHIV. Among 11 male respondents, six were international migrant workers who lost their international jobs immediately after being diagnosed with PLHIV. After returning to Bangladesh, they started working in Bangladesh to cope with the severe financial crisis due to increased ART costs. Three of four female respondents were widows; their husbands were international migrant workers and died due to HIV after returning to Bangladesh. Among all 15 respondents, the early diagnosis was in 2004, and the early start of ART was in 2005. Nine out of 15 respondents started ART immediately after diagnosis. At the time of data collection, though all were under ART, they discontinued their treatment at different phases as they faced diverse sorts of barriers presented in the result section under different themes and sub-themes. This leads to the development of a thematic framework (Fig 1) after merging the related sub-themes under three major themes: individual, community, and institutional levels, according to the health behaviour change socio-ecological model [22].

### Individual-level

We found three sub-themes: forgetting the doses, being away from home, and financial barriers, which acted as barriers to continuing ART at the individual-level; these sub-themes are related to the respondents’ psychological behaviours and socio-economic conditions. In addition, we found two sub-themes that acted as a motivation to overcome the barriers to continuing ART.

#### Forgetting the doses

Less than half of the respondents reported periodic forgetting to take their regular doses. We investigated the underlying reasons for forgetfulness and found that the study respondents intentionally did not want to forget to take medicines. Initially, they found it challenging to maintain a regular schedule for taking medicine, especially in the case of those doses that were supposed to be taken before bedtime. They forgot to take medicines due to circumstantial contexts rather than cognitive impairment, such as falling asleep or getting drunk while being an alcoholic.

*“I was supposed to take medicine after my dinner*. *It happened two times that I forgot to take medicine*. *Actually*, *I was lying on the bed after dinner and fell asleep*.*”* [Male living with HIV, 45 years old]

*“It was like I thought that I had my medication and slept*. *When I realized that I had not taken medicine*, *it was very late to take the missed dose*.” [Male living with HIV, 40 years old]

“*I am an alcoholic; I just forgot to take medicine whenever I was drunk*.” [Male living with HIV, 41 years old]

One of the respondents reported an incident of an overdose of the medicines.

“*I never missed*, *but I took an overdose*. *I have been prescribed one medicine for the morning and two for the night*. *I forgot to take the medicine at night*, *instead I took it in the morning*.” [Female living with HIV, 36 years old]

#### Being away from home

Besides forgetting, being away from home also acted as a barrier to treatment adherence. Two male respondents reported forgetting to carry the medicine while away from home, for work or for other issues.

“*Sometimes*, *if I go somewhere for work*, *then I miss the dose because I usually forget to carry the medicine*.” [Male living with HIV, 37 years old]

“*Yes*, *I missed it*. *When I was outside of my home once or twice*, *at that time*, *I missed the medicine*.” [Male living with HIV, 45 years old]

#### Financial barriers

Despite free ART, nearly half of the respondents mentioned financial constraints as barriers to treatment adherence. Due to financial constraints, they could not buy supplementary medicines when those were not available at the ART centre. The following quotes illustrated more about their sufferings related to economic barriers:

*“We used to get many other supplementary medicines*, *like antihistamine*, *vitamin*, *calcium*, *and other supplements*. *However*, *nowadays*, *ART centres do not provide these (supplementary medicines)*. *If I asked for it*, *they (healthcare providers) would ask us to buy it from outside and I could not buy it as I didn’t have money*.” [Male living with HIV, 45 years old]

They faced difficulty visiting the ART clinic when they could not manage transportation costs to check whether medicines were available as the ART centre was far from their houses. Due to the financial catastrophe, a few respondents could not buy medicines if those were not available in the government’s ART centres due to lack of or late supply. In Bangladesh, most of the medicines related to ART are enlisted for free dispense. Doctors also prefer that PLHIV should not discontinue the medication to avoid drug resistance; that is why they suggest that PLHIV should buy medicine from outside for a few days until supply is available in government centres.

“*Most of the PLHIV are not financially stable*. *For them*, *it becomes a hazard to come (to the ART centre) from distant places to take medicines*. *So*, *they just give up*.” [Female living with HIV, 36 years old]

*“Sometimes*, *we do not get the medicine on time*, *so we cannot take medicine*.” [Male living with HIV, 37 years old]

Though the ART program is accessible at free of charge in Bangladesh, it is the PLHIV’s responsibility to do the CD4 count test. The WHO guideline suggests doing the CD4 count test every six months, whereas, in our study, we found that respondents cannot do this costly test regularly every six months due to financial limitations.

*“I tested my CD4 only once yearly (instead of every six months) because the test is costly*.” [Male living with HIV, 46 years old]

In addition, respondents provided insights about their motivation to overcome ART adherence-related barriers. The positive approach toward their life and family support motivated them to overcome the barriers.

#### Positive family support

Two respondents mentioned their family support as a positive factor that helped them to adhere to their treatment. They stated that they get mental strength and self-motivation to fight against their PLHIV condition as they receive family support. Family support, especially from parents and siblings, increased their willingness to live and maintained a strict adherence to treatment.

“*We have to generate mental strength and self-motivation*. *If I lose my willingness (to live)*, *then no one can make me feel it*. *A PLHIV’s main support should come from family; after that*, *medicine and regular maintenance (can help them)*. *If the family support is missing*, *then there will be no or fewer chances to continue this life though people’s birth and death are on the hand of only Allah (GOD)*.” [Female living with HIV, 38 years old]

“*I actually did not face any problem*, *as my parents knew*. *Especially my mother*, *she always reminds me to take medicine all the time*.” [Male living with HIV, 35 years old]

#### Positive approach toward life

Despite having a compromised life, some PLHIV still have a positive approach toward the disease and their lives. Over the years, they fought against all the obstacles and gained self-strength and confidence. Also, they could make their close ones to understand the fact of PLHIV.

*“Yes*, *sometimes I feel that I am living a pathetic life*, *and sometimes I want to quit*. *But when I look at people living on the footpaths (pavement*, *a narrow space for pedestrians to walk)*, *I feel motivated to live again; they are homeless*, *but I have a house……*..” [Female living with HIV, 38 years old]

“*Now I have that much confidence to reply (to) them; I said that my disease would not cure permanently*, *and I have to take medicine lifelong*. *Earlier*, *even my relatives misbehaved with me*, *did not like to attend my family gatherings*, *or did not like my companion*. *But*, *now they invite me to visit their home and have a meal with me as well*.*”* [Male living with HIV, 45 years old]

### Community-level

At the community-level, we found stigma as the most compelling study finding, which almost all study respondents commonly reported. The theme “stigma” emerged as one of the challenging barriers to ART adherence from the psycho-social perspective, and it consisted of six sub-themes: discrimination, fear of disclosure, depression, misconception, rejection, and lack of awareness. All these obstacles made the PLHIV’s situation more vulnerable to treatment non-adherence.

#### Discrimination

Nearly all our study respondents shared their experience of suffering from discriminatory acts at every stage of their life; they were discriminated against either at home by their family members or relatives or in society by neighbours and even by colleagues or employers in their workplaces. They were also discriminated against by medical personnel from where they had to take treatment. In most cases, this discrimination destructively affected their treatment adherence and the quality of their daily life, including financial stability. Because of discrimination, they had to remain jobless or deprived of their assets, could not stay either at their own or relatives’ houses, and even remained scared of life-threatening.

*“In 2010*, *I was admitted into IDH (Infectious Disease Hospital*, *a government hospital located in the capital Dhaka*, *mainly treating different kinds of infectious diseases*, *including HIV)*. *There was a nurse who used to live in my brother’s house*. *She told my sister-in-law that she should not allow a PLHIV to live in her house……*. . . *That incident hurt me very badly because I could not live without them*.” [Male living with HIV, 55 years old]

“*I went to a doctor in my village*, *and he took my blood sample in order to know what happened to me*. *After three days*, *he did not even allow me to enter his room*. *Not only that*, *he informed one of my villagers*, *and that villager informed the whole village about my PLHIV status*. *The villagers started to threaten me and asked me to leave my village or else they will kill me………*.. *I was afraid to go there (my own village/home town) as my family was also being threatened (by villagers)*.*”* [Male living with HIV, 40 years old]

One of the study respondents had been terminated from the job immediately when the employer came to know about her PLHIV status, and they (the organization) did not show any valid reason but made the respondent bound to quit the job.

“*I worked in a government office for nine days; after a few days*, *a few of my neighbours who knew about my problem (PLHIV status) informed my boss about it*. *Then my boss fired me immediately*. *At that time*, *I did not know (about PLHIV-related human rights) as much as I know now*. *Otherwise*, *I could defend my position to my boss*.” [Female living with HIV, 25 years old]

#### Fear of disclosure

Fear of disclosure emerged as another prominent sub-theme, followed by discrimination. Most of the study respondents shared their views about not disclosing their PLHIV status, as they were afraid of being rejected by society and even by their close family members. They also had concerns about being discriminated against and facing uncomfortable situations or any humiliation by their family or society. As a result, they could not openly visit a doctor or ART centres for treatment purposes.

“*I am a counsellor*, *but still*, *I am scared of if the society knows that I am a PLHIV*, *how they will react*, *what will happen to my children…*. *will society accept them (children) or not*.*”* [Female living with HIV, 38 years old]

“*I keep this matter secret because I do not want to find myself in an uncomfortable situation*. *I do not know what will be their (family members’) reaction after knowing it (PLHIV) …*. . . *That is why I sometimes hesitate to take medicines in front of them*. *Often I take the medicines after they fall asleep*.*”* [Male living with HIV, 41 years old]

*“General people never like PLHIV*. *I never shared my (PLHIV) status with anyone*, *not even with (my) close ones*. *Because I think if they know about it*, *they will hate me and not accept me*, *even may leave my companion*. *People fear of being treated disrespectfully*, *that is why ……sometimes they even hesitate to access the healthcare facility; all these are due to the outlook of our society*.*”* [Male living with HIV, 39 years old]

The study respondents expressed their concern about the new PLHIV and mentioned that they were also afraid of social rejection and harassment, thus avoiding visiting the ART centre.

“*I think new PLHIV who are not coming for treatment regularly mainly due to fear of disclosure*.” [Male living with HIV, 41 years old]

#### Depression

Almost half of the respondents reported suffering from depression due to discrimination, fear of disclosing their PLHIV status, and social rejection. Sometimes, their own family’s discriminatory behaviour causes mental devastation, and the sufferer gradually starts to think of quitting treatment or even committing suicide. Such responses were thus coded under depression.

*“My sister-in-law spreads the news of my PLHIV status to others without (taking) my consent*. *This incident made me depressed*. *I thought that I was suffering due to my fault*. *Furthermore*, *now (almost) everyone knows about it*, *and they started to hate me*. *They will never accept me as they used to*. *I lost the hope to live and thought of committing suicide*.” [Male living with HIV, 55 years old]

Being treated differently affects the respondents’ mental health, and ultimately, their treatment-seeking behaviour. General people always consider that PLHIV has a bad moral character, that is why they hesitate to help them. Respondents further explained that from the fear of being humiliated by others, they not only suffered from depression; they also could not lead a normal life like a healthy (living without HIV) person and could not seek treatment like others who are not living with HIV. Sometimes, they start to have depression at a very early stage, right after being diagnosed with PLHIV, which might lead to severe depression later.

“*I always hesitate to touch the belongings of others*. *Sometimes*, *I wanted to help others or share my food*, *but I could not do it as I hesitated whether people would behave normally with me or accept my food and help*. *I am afraid of the discriminatory act because it will make me depressed*.” [Female living with HIV, 38 years old]

“*I went there (hospital) and did all the check-ups*. *Then he (the doctor) came to my home after three days*. *He told me that I was PLHIV*. *I requested him not to tell anyone about this*. *After that*, *I was mentally shattered and decided to leave home …*. *and decided not to keep in touch with my family*. *I stopped taking ART…*.. *I was in such a depressive condition that it took almost three months to bring me back to normal through counselling*.” [Male living with HIV, 41 years old]

#### Misconception

Misconception about the mode of transmission of HIV was found to be another sub-theme that leads to stigma among PLHIV. Most of the respondents experienced a traumatized mental situation due to having a misconception of the general people regarding the mode of transmission. We also found that general people have a misconception about PLHIV’s external facial or physical appearance; even general people think that PLHIV might look like an animal, as quoted by the following respondents:

“*General people think that PLHIV looks like a monkey or other animals*, *that is why they get curious to see a PLHIV*. *Sometimes people get mad at us and always try to avoid mixing with us*.” [Male living with HIV, 35 years old]

“*I did a show (TV program)*. *Suddenly all of their staff came to see me as a PLHIV*. *They said that they wanted to see how PLHIV looked like…*. . .” [Male living with HIV, 41 years old]

General people also had the wrong idea about the transmission mode of HIV infection; they think it can be spread through touching or body fluid (saliva) released from the mouth. The following quote illustrates the knowledge gap and misconception about HIV’s transmission mode.

“*She (sister-in-law) told other people not to live with me as I am a PLHIV*, *which is a bad disease*. *She also told them*, *“never eat in his house*, *never allow him to touch your babies; if you do so*, *then you and your baby will also be infected*.” [Male living with HIV, 55 years old]

Even respondents mentioned that some people think that being a PLHIV is a crime, and the law enforcement team should shoot that person down.

“*When I returned to my hometown again (with my husband*, *who is a PLHIV)*, *some of our neighbours warned us not to disclose it (PLHIV status) to others because they said that police would shoot my husband because of his PLHIV status*. *I was so scared at that moment that I also lied to my parents by saying that my husband was suffering from liver problems*.” [Female living with HIV, 38 years old]

#### Rejection

Rejection was another important sub-theme that influenced PLHIV to be stigmatized. Almost all respondents mentioned rejection as a barrier. Their spouses and parents rejected the respondents because of their wrong perception of HIV infection.

“*One day*, *I went to my parent’s home to meet my mother*. *She suddenly wanted to know who am I*. *I replied*, *“Mother*, *I am your daughter*.*” She told me never to revisit her*. *Actually*, *neighbours criticized me badly over there*, *and she (mother) thought that my siblings would also be infected if I went there*. *I still did not tell them the reason for my husband’s death (he was also a PLHIV); I fear they (my family*, *friends*, *relatives) will reject me*.” [Female living with HIV, 38 years old]

“*Society considers us as bad people*. *They think that PLHIV is like a virus*, *which is destroying society*.” [Female living with HIV, 36 years old]

Even our study respondents were rejected by healthcare providers (HCPs). Whenever HCPs could find out the PLHIV status among patients, they directly denied giving treatment to that particular patient. The respondents (“Male living with HIV, 37 years old”) shared an incident that happened to one of his peers where the HCPs did not want to do any surgery when they came to know about his PLHIV status.

“*I know a person who was a PLHIV*. *He had pain in his teeth*, *so he went to many dentists*, *but nobody wanted to help him to pull out his teeth due to his PLHIV status*. *……………Then*, *he went to a dentist and got the treatment without informing him about his PLHIV status*.” [Male living with HIV, 37 years old]

#### Lack of awareness

More than half of our respondents shared their opinions about the importance of raising awareness among the general people so that the misconception regarding the mode of transmission and discrimination against PLHIV could be removed. Otherwise, they may continue to face social distress and hatred. To elaborate on this sub-theme, the following quotes were cited by two respondents “Male living with HIV, 45 years old” and “Male living with HIV, 48 years old”:

*“I think we should raise the awareness among the general people about the (mode of transmission of) HIV/AIDS*. *Social awareness can remove those obstacles which a PLHIV faces*. *Moreover*, *most of these barriers lead to social distress*, *that is why we do not want to share (our PLHIV status) with people as they will treat us with hatred*.*”* [Male living with HIV, 45 years old]

“*I think there is some lacking (of awareness)*. *If more awareness programs could be conducted*, *then people would have more knowledge about the disease and could take it (HIV) as a common disease like diabetes*.” [Male living with HIV, 48 years old]

### Institutional-level

We found a set of barriers at the institutional-level that interfere with ART adherence. This theme consisted of three major sub-themes: medicine-related barriers, treatment-related barriers, and healthcare facility-related barriers.

#### Medicine-related barriers

Side effects of medicine and insufficient or irregular medicine supply were considered under the sub-theme “medicine-related barriers”. A side effect is an unwanted outcome related to every medicine; this can happen even with prescribed medicines or over-the-counter medicines. Two respondents mentioned side effects as one of the barriers to treatment adherence. Most of the side effects were manageable, like rash; a few side effects may become severe, like forgetfulness. Side effects might appear immediately after initiation of ART as the following quotes explain,

*“When I first started the ART*, *there was a rash in my leg; then the doctors gave me some antibiotics to cure that rash*.” [Female living with HIV, 25 years old]

“*No one likes to take medicine as every medicine has side effect*. *I am having a problem with remembering things*. *It started after I started taking ART*. *That is why sometimes I felt like to discontinue (it)*.*”* [Male living with HIV, 37 years old]

Three respondents mentioned how the irregular medicine supply at the ART centre affected their treatment adherence. They complained about the frequent shortage of medicine stock. It might have happened due to mismanagement in regulating the medicines’ stock. They mentioned that they usually receive medicines for two months according to the government policy, but they could not receive them regularly for a couple of months due to a lack of supply. They mentioned that they faced this kind of irregular medicine supply situation mainly in the ART centre located inside the government hospital. For this reason, “Female living with HIV, 38 years old” had to discontinue one of her medicines for two months, and “Male living with HIV, 55 years old” also shared a similar kind of situation below,

*“I have been taking three medicines (named Ritocom*, *Abacavir*, *Lamivudine) since the treatment was started (the Year 2009)*, *but after a few months*, *I had to stop taking one medicine (Ritocom) because that medicine was out of the market…… After the availability of that medicine*, *I started taking that (medicine) again*.*”* [Female living with HIV, 38 years old]

*“Nowadays*, *we do not even get medicines regularly*. *Sometimes*, *they give medicines only for 10 days*, *sometimes for one month or two months*. *When we wanted to know the reasons*, *he (pharmacist) replied that the ART centre was running out of medicines…*. . . *Previously*, *we did not have to face this kind of problem; now it has become a constant problem*.*”* [Male living with HIV, 55 years old]

#### Treatment-related barriers

Lack of proper counselling, improper treatment and management, and skill gaps in diagnosis were considered under the sub-theme “treatment-related barriers”.

Counselling is an essential component of HIV treatment. PLHIV needs counselling to reduce their mental stress and strengthen their self-confidence. Slightly more than half of our respondents shared their perceptions about the importance of counselling from different points of view:

“*If someone has HIV*, *s/he normally goes through mental stress*, *so they must attend the counselling sessions*. *Then*, *they could be mentally stronger*. *I think sometimes this counselling works better than the medicine*.” [Male living with HIV, 37 years old]

They also mentioned that the counselling was equally crucial for new and existing PLHIV because both needed different kinds of mental support. In the case of new PLHIV, counselling helped them to cope with this adverse situation and prepared them to fight against disease-related mental stress by sharing the proper knowledge. Counselling also motivated and inspired the new and the existing or old PLHIV (who have been taking ART for years) to adhere to their medicines.

“*New PLHIV thinks that this virus will kill them eventually*, *and thus they lose the hope of living*. *They also do not know where to go for treatment*, *how to take medicine*, *how they will share (their disease status) with their family*, *what society will think about them*, *and how they will be healthy again*. *So*, *it takes time to understand and remove the fear of HIV/AIDS*. *So*, *I think proper counselling is crucial to realize that this is not their fault; they need time to cure*. *Some PLHIV needs motivation as they have been taking this ART for the last 5 to 6 years; they just need to boost up their will power or inspiration so that they can continue their medicine*.” [Female living with HIV, 38 years old]

Regarding proper counselling, half of the study respondents showed their concerns about not having the proper counselling. Mainly they were not getting sufficient time and mental support from the HCPs, who may help them to reduce their stress and treatment adherence. Respondents “Female living with HIV, 25 years old”, “Male living with HIV, 37 years old”, and “Male living with HIV, 39 years old” strongly agreed upon this issue; they had more concerned about newly PLHIV who will suffer more due to lack of proper counselling and follow-up.

“They (new PLHIV) do not get proper counselling; mainly, the new PLHIV are suffering as they do not know the proper way to take the medicines.” [Female living with HIV, 25 years old]

“Honestly, they (govt. hospital) do not provide (to us) counselling.” [Male living with HIV, 39 years old]

“Now we take medicine from BSMMU (Bangabandhu Sheikh Mujib Medical University, a government graduate medical university, and hospital which has the main ART centre for PLHIV), but they (also) do not give (us) any counselling. That is why it is challenging for the new (PLHIV) members (to get any counselling). Earlier, when we took medicine from AAS (an NGO), we always attended counselling sessions…..new members are also frustrated.” [Male living with HIV, 37 years old]

Regarding improper treatment and management by HCPs, one of the study respondents shared his bitter experience. Over time, he found that due to lack of proper treatment and management, he transmitted the HIV infection to his daughter by donating his blood.

*“I had a daughter*, *who had thalassemia (an inherited blood disorder)*, *I used to give her blood frequently*. *Then in 2010*, *once my daughter’s health condition declined suddenly*. *I took her to India (the neighbouring country for better treatment)*. *While giving blood to my daughter*, *they (Indian doctors) diagnosed that I am PLHIV*. *If in Bangladesh*, *they (Bangladeshi doctors) could do the test before I gave blood to my daughter*, *then my daughter would not be infected because of me*. *She might still be alive*. *Now sometimes I feel that I am responsible for her death*.” [Male living with HIV, 46 years old]

Our respondents also shared their unpleasant experiences due to the presence of skill gap in diagnosis. They attempted to diagnose their disease in the initial stage, earliest in 2005 when in Bangladesh the healthcare providers and consultants faced difficulty in diagnosing HIV/AIDS in the first place due to a lack of proper knowledge and skill. As a result, the respondents could not start their treatment as early as possible. In some cases, respondents had to spend almost all of their wealth and savings to get the proper treatment as the consultants suggested doing many tests to make the proper diagnosis which were very expensive. The following quotes illustrate more about this scenario,

“*I spent almost 45 lacs (USD 56*,*250) for the treatment*. *I sold almost everything (assets and properties) to arrange this money; however*, *(I) did not find the right treatment at that moment*.” [Female living with HIV, 38 years old]

*“I spent almost all of my savings*, *like 14 lac (USD 17*,*500) taka*, *for my treatment*. *Unfortunately*, *I did not get any results*. *I went to costly hospitals too*, *but they were also unsuccessful in diagnosing the disease*. *………………*.*Then I went to a well-known doctor of Bikrompur (close to my hometown); he was my last hope*. *In addition to his consultation fee*, *I spent 99000 (USD 1238) taka in only six days as he gave me to do almost all the possible tests*. *Nonetheless*, *he could not diagnose*. *Then lastly*, *he asked me to do the HIV test*.” [Male living with HIV, 55 years old]

#### Healthcare facility-related barriers

This sub-theme includes the perspectives of facility’s infrastructure and healthcare provider. Regarding the ART centre’s infrastructure, most of the study respondents expressed strong negative opinions of the unsuitable treatment-seeking environment, mainly about sitting arrangements. Additionally, the ART centre was reported to be overcrowded. This unsuitable environment demotivated them to visit the ART centre. The following quotes narrated the respondents’ view in detail:

“*I do not think that the ART centre’s environment (physical infrastructure) is suitable for us*. *The room is small and very congested*. *They (health facility cleaners) do not clean the place regularly*”. [Male living with HIV, 45 years old]

*“………*.*… hospital physical arrangement is not comfortable enough for PLHIV … the hospital does not have enough sitting arrangements or facilities*. *I will mark it 0 out of 100*. *In summer*, *people suffer a lot due to heat*.” [Female living with HIV, 36 years old]

Regarding HCPs, less than half of the study respondents shared their concerns about not having any specific doctors or specialists in the ART centre for PLHIV. The following quotes highlighted how the generalization of treatment due to the lack of specific doctors for PLHIV affects their treatment adherence, especially in the government hospital.

“*Sometimes*, *doctors are very busy with others (non-PLHIV); that is why we have to wait for long*. *Another issue is that there is no (specific*, *HIV/AIDS specialized) doctor who can provide consultation*, *especially for PLHIV like us*. *They provide us consultation and treatment (simultaneously) along with others (non-PLHIV)*.” [Male living with HIV, 45 years old]

“*The same doctors are consulting different (kinds of) patients like TB or other infectious diseases in the same place at the same time*. *That is why they do not have enough time to properly consult a PLHIV*.” [Male living with HIV, 41 years old]

Most of the respondents reported a long waiting time as one of the barriers. This has happened because doctors were not punctual to join their workplaces and a lack of sufficient HCPs leads to a high doctor-patient ratio. Their suffering to continue treatment is illustrated by the following quotes,

“*Normally*, *I have to wait at least 30–45 minutes*. *If I go to BSMMU (Bangabandhu Sheikh Mujib Medical University*, *a government graduate medical university and hospital which has the main ART centre)*, *it takes more time as you know (that) BSMMU has recently become the main (ART) centre*. *So*, *there are many patients at a time*, *and also there is a lack of staff*, *I feel*.” [Male living with HIV, 41 years old]

*“…*. *we need to queue for consultation and most of the time*, *doctors are not (at their chambers) on time*. *So*, *we need to wait for 45 minutes to 1 hour at least to meet a doctor*.” [Female living with HIV, 38 years old]

In addition to the long waiting time, the respondents also mentioned that HCPs were not friendly enough. So, they hesitated to share their clinical problems with HCPs.

“*PLHIV must wait for a long time to get their consultation*, *treatment or medicines*. *The doctors do not follow the official rules and work as they wish*. *For this*, *many PLHIV coming from different parts of the country face many troubles*. *The staff is not friendly as well*.” [Male living with HIV, 40 years old]

“*I do not find anyone over there who can listen to my sufferings or health problems*. *I do not feel comfortable; so*, *I do not try to share anything (health problems)*.*”* [Male living with HIV, 35 years old]

Concerning trust and confidentiality, more than half of the study respondents expressed mixed reactions. Some of them felt that HCPs were not trustworthy and they could even leak their information. They also revealed that the place is congested and located in an open place; therefore, it was impossible to maintain privacy.

“*Sometimes*, *they (nurses) discuss with their colleagues or other persons about us as we are PLHIV; we understand it as they often point towards us when they talk among themselves*. *Moreover*, *it is an open room in the outdoor section*, *so it is almost impossible to keep all the information private*.” [Male living with HIV, 39 years old]

“*I feel that they can leak our confidential information*. *They (doctors and nurses) can share (it) with other staff regarding us and treat us differently*. *It is almost impossible there (to ensure privacy)*, *as the place is very congested and small*. *You can even overhear the conversation from the counselling room*.” [Male living with HIV, 55 years old]

On the other hand, other study respondents believed that their information was safe with the HCPs; they could have faith in them. The following quotes explained the positive perspective of the study respondents.

“*I think they (HCPs) can maintain confidentiality about my problems*. *And*, *it does not matter to me if they leak my confidential information (to others)*.*”* [Male living with HIV, 46 years old]

*“They always tell us that they would not disclose any of our information*. *So*, *I believe that my treatment history or information is safe with them (HCPs)*.*”* [Male living with HIV, 48 years old]

Similar to trust and confidentiality, more than half of the study respondents also gave a mixed reaction related to “respect”. Some respondents mentioned that the HCPs maintained a distance from them as they knew about their PLHIV status, and some respondents believed that due to lack of training, the HCPs’ behaviour was not proper towards the PLHIV.

“*Yes*, *sometimes they (HCPs) try to avoid or keep distance after knowing that I’m a PLHIV*.” [Male living with HIV, 48 years old]

“*No*, *as they are not well trained*, *their approach is not proper*.” [Male living with HIV, 37 years old]

On the contrary, some of the study respondents shared that they were treated with respect by the HCPs always as cited below,

“*Yes*, *they do*. *Especially Dr*. *X is very humble*, *and he always behaves like our family members or close one*.” [Male living with HIV, 45 years old]

“*Yes*, *I get the proper respect from them*.” [Female living with HIV, 25 years old]

Besides, “Female living with HIV, 38 years old” shared that she was a victim of dishonour when she was misbehaved by one of the government hospitals’ doctors. That misbehaviour made her husband lose hope for his life; he became depressed after that incident and wanted to discontinue his treatment.

*“When my husband was sick*, *I took him to CMH (Combined Military Hospital*, *Dhaka) for a CD4 test*. *I had to wait with my husband for 24 hours at the outdoor wing (department)*. *They (doctors) awfully misbehaved with us…*.. *The doctor screamed at me by saying that “you know your husband has AIDS; why did you bring him here*? *There is nothing that I can do for him*.*” Upon hearing this*, *my husband cried and requested me to take him away from there; he did not want to continue any treatment…… he just prayed for his death*.” [Female living with HIV, 38 years old]

## Discussion

HIV-related morbidity and mortality declined due to ART, which improves PLHIV’s quality of life. HIV/AIDS converts to a well-regulated chronic disease instead of a terminal disorder where ART has been made extensively accessible, and high levels (at least 95%) of patient adherence have been guaranteed [23]. Optimal treatment adherence indicates patients’ ability to take medicines on time and at prescribed frequencies according to the treatment plan, including food and other medication restrictions [24]. This study identified the multi-level barriers to ART adherence to suggest how to overcome those barriers and ultimately end HIV/AIDS in Bangladesh.

Evidence suggests that as a potential psycho-social barrier, stigma is negatively related to self-reported ART adherence among PLHIVs, who are often stigmatized by the general population. HIV-related stigma is also related to discrimination and affects the mental health and quality of life of PLHIV [25–28]. Similar to multiple study findings [28–35], we also found stigma as the most frequently reported prominent barrier to ART adherence, mainly in the form of discrimination and rejection due to misconception and lack of awareness, leading to fear of disclosure and depression. This discrimination and rejection destructively affect their treatment adherence and quality of life, including financial stability.

There was significant evidence that the discriminatory act eventually led to fear of disclosure due to harassment and rejection [36–42]; we also observed the same among our study respondents, which seriously affected their treatment adherence. Similar to other study findings [32, 43, 44], our study respondents showed their concerns about the new PLHIV, as they may be afraid of visiting ART centre to avoid social discrimination related to social isolation and harassment. Our respondents assumed that this could be one of the main reasons for the delay in the starting of ART by the new PLHIV. We also investigated to identify the underlying causes of social stigma and discrimination. We found that misconception and lack of awareness about the transmission mode are the main reasons for social stigma and discrimination. The Bangladeshi general population has a misconception that HIV can be spread through touching, talking, or mixing up with PLHIV, and PLHIV may look like monkeys or other animals. This misconception about the transmission mode of HIV infection persists among the general population of other countries like China, Nigeria, and Malawi due to a lack of adequate knowledge and health education interventions [45–47].

Our study findings related to poor treatment adherence depending on PLHIV negative emotional aspects like anxiety and depression is also reflected in similar studies conducted in Ethiopia [48, 49]. Furthermore, our study respondents highlighted their encouraging family support as a low-cost source of self-motivation and positive emotional strength, which is needed to overcome the emotion-related barrier to initiate the treatment and maintain adherence strictly. A similar study finding is illustrated by Ayer et al. (2016), who found positive family support as one of the motivating factors to maintain the Nepalese patients’ mental strength and encourage the patients to attend the health facility and continue the treatment [50].

The discriminatory act also prevailed among the Bangladeshi healthcare providers (HCPs) while providing treatment and consultation at the healthcare facility due to a lack of comprehensive HIV-related training to change their negative attitude. HCPs’ negative attitude is also linked to trust and respect, and more than half of the respondents mentioned that they could not trust HCPs. Hence, they hesitated to depend on them and disclosed their confidential information for proper treatment. A previous study conducted among HCPs in Bangladesh also found similar findings, i.e., the negative attitude of HCPs toward PLHIV hampering their treatment adherence [29], i.e., the situation did not change much to improve treatment adherence. Our study findings highlighted the importance of strengthening comprehensive HIV-related knowledge education to change the attitude of Bangladeshi HCPs. The stigmatizing attitudes of HCPs toward PLHIV are also predominant in other countries like China, Lao PDR, Vietnam, Brazil, Indonesia even in the Netherlands, which critically affects ART adherence [51–58].

Besides healthcare providers’ negative attitudes, we also identified several barriers related to healthcare providers and facilities. Lack of proper counselling, especially in government ART centres, is one of them, and it is essential for treatment adherence. Supportive counselling sessions, either one-on-one or group counselling, are effective to ensure long-term ART adherence [59]. Our study respondents, who received supportive counselling from non-government ART centres’ senior and trainer counsellors, learned to be self-confident; thus, they become self-motivated and optimistic about their lives despite facing discrimination and rejection in society.

We found that the study respondents suffered from being diagnosed on time due to a lack of specialized training on HIV/AIDS among Bangladeshi healthcare professionals; as a result, PLHIV could not receive timely effective treatment and management. It also affected them financially as PLHIV had to visit different doctors and to do multiple tests before being diagnosed. Azim et al. (2008), also presented the similar findings on insufficient trained HCPs in Bangladesh by highlighting their lack of proper training to diagnose and treat PLHIV [12].

In order to address this issue, the Bangladesh government has undertaken some of the strategies in the latest National Strategic Plan (2018–2022). One of the essential strategies focused on the capacity development of the healthcare professionals to provide comprehensive treatment for PLHIV. In addition, the Bangladesh government decided to establish a well-equipped specialized healthcare facility to manage complicated HIV patients to end AIDS by 2030 [13, 60].

We identified several healthcare facilities-related barriers; among them, the most common is an unfavourable treatment-providing environment. This is due to the small room size where treatment, consultation, and laboratory testing were provided simultaneously. Hence the clinical site was highly congested and badly affected to maintain the patients’ privacy and confidentiality. A similar situation has been found in the study conducted in African countries like Mozambique, Uganda, and Malawi [61–63].

In addition, the majority of respondents mentioned long waiting times as one of the barriers; this happened mainly due to the lack of sufficient ART-trained physicians and consultants and the irregular presence of healthcare providers in the ART centre as they do not come to work on time. Besides, there was a lack of proper sitting arrangement; hence, the study respondents faced difficulties taking rest while waiting in a long queue for treatment and management. This discouraged them from visiting ART centres, which affected their treatment adherence. The long and uncomfortable waiting period was also identified as an ART adherence barrier by Becker et al. (2020), Chirambo et al. (2019), and Hardon et al. (2007) [64–66].

Due to a shortage of HIV specialists, PLHIV had to seek treatment from general healthcare providers who simultaneously provided treatment to non-PLHIV. It affects the quality of service, mainly the proper counselling, due to a lack of sufficient time and proper training among the general healthcare providers. There is significant evidence of the positive association between proper counselling and ART treatment [67–69]; hence, the Bangladesh government needs to emphasize strengthening proper counselling in HIV management.

Another study finding is improper inventory management. Our study respondents mentioned that the ART centre frequently ran out of stock (ART-related medicines). It might be due to mismanagement in regulating the stock of medicines. As a result, many PLHIV missed regular doses and failed to adhere to the treatment. Similarly, a study conducted in Kinshasa also found frequent stock-outs of ART-related medicines in their healthcare facilities, which increased the risk of HIV transmission and viral resistance [70]. ART-related medicine stock-out is also identified as a significant barrier to treatment adherence in several African countries and India [71–75]; hence, this inventory mismanagement issue needs to be addressed appropriately to ensure the continuation of treatment.

Our study respondents experienced rash and trouble in memory to remembering any object or incident as a side effect of ART. The side-effect is also considered a significant barrier to ART adherence in the studies conducted in Ghana [76], Ethiopia [77], Iran [78], and China [79, 80]. However, the Thai study population did not consider side effects a severe barrier to discontinuing their ART as they considered ART a life-saving measure for PLHIV [81].

In addition, we found that individual-level barriers, like forgetting to take medicines, being away from home, and financial barriers, negatively influence PLHIV treatment adherence. Similarly, multiple studies identified forgetfulness as one of the common barriers to ART adherence due to different underlying reasons [76, 82–85]. Studies conducted in African countries found that alcohol abuse leads to forgetfulness of ART adherence among African women [64, 83]. Similar to our study, related studies also found that respondents forgot to take medicine when they were busy with work or social activities outside their homes [76, 82, 84].

Furthermore, economic barriers negatively influenced the treatment adherence of our study respondents. Most of our study respondents were from low or low-middle-income families, and many were unemployed. Due to financial constraints, they faced difficulty buying ART-related and supplementary medicines if needed. Although PLHIV is supposed to receive ART-related medicines entirely free in Bangladesh, sometimes PLHIV has to buy ART-related medicine from outside drug stores at a high cost due to a lack of regular supply. Financial constraint is a significant barrier to buying those expensive ART-related medicines to ensure their treatment adherence. Due to financial constraints, our study respondents faced difficulty purchasing supplementary medicines, which are not available at the ART centre. They also faced difficulty visiting the ART clinic when they could not manage transportation costs and sometimes could not do CD4 tests every six months due to financial limitations.

The financial limitation is also a notable obstacle to poor treatment adherence in other studies conducted in low or lower-middle-income countries [61, 86–89]. Similar to our study findings, African studies found that transport costs challenge ART adherence [66]. Especially the poorest PLHIV living far from the ART centre had to be worried about their transportation costs; sometimes, they failed to visit the ART centre on time due to financial constraints [90–92].

Bangladesh recognized HIV/AIDS as one of the emergent health and social problems and formed the National AIDS Committee in 1985 before the first case was detected in 1989 [13].

Since then, the Bangladesh government has actively taken multiple initiatives to control the HIV epidemic. The Bangladesh government has developed a comprehensive policy and framework focused on providing ART for PLHIV and HIV/AIDS prevention programs for the general population. Nevertheless, only two national pharmaceutical companies produce six types of ART-related medicines, and limited ART-trained clinicians are available [12, 93]. In addition, according to the Anderson-May equation, it has been found that the “counselling for PLHIV” intervention was not implemented effectively [15]. Hence, actions should be undertaken to ensure ART-related medicines by the trained service providers and strengthen the HIV counselling service to cover the growing number of PLHIV to reach close to 100% ART coverage.

The Bangladesh government needs to intensify the public awareness and behaviour change communication (BCC) campaigns of HIV/AIDS, including Information Education Communication (IEC) activities like mass media campaigns, to remove the misconception about the transmission mode of HIV. Similar to other studies conducted in Bangladesh [94–96], we also identified the need to strengthen the national awareness-raising campaigns focusing on the urban and rural population to have the greater impact on reducing the HIV-related stigma and discrimination, which delay HIV testing and uptake ART.

The Bangladesh government has already initiated the necessary steps to eliminate HIV- related stigma and discrimination by promoting laws and policies and developing a time-bound action plan that includes training manuals and toolkits [13]. However, the implementation of the action plan needs to be reinforced and strengthened by conducting community-based interventions. Effective advocacy is required to implement the policy and interventions related to increasing access to HIV prevention and treatment services.

Systematic reviews suggested that PLHIV needs frequent reminders through SMS messages and proper counselling on the importance of taking medicine on a regular basis to overcome the most cited barrier, “forgetfulness” [1, 97]. Regular mental health screening of PLHIV and ensuring the establishment of providing mental support through supportive counselling and group psychotherapies are the suggested corresponding interventions against negative emotional aspects like depression [1, 98]. Bangladesh policymakers can include these interventions to improve the ART adherence rate, significantly strengthening the counselling services both at the government and non-government ART centres, which play a significant role in treatment adherence, reduce mental distress, and give self-motivation to fight against stigma and discrimination.

We identified barriers at the individual, community, and healthcare institutional levels and found how they affect ART adherence. Literature suggests a comprehensive model to change health behaviour at multiple levels; this model provides practical recommendations for HIV prevention and AIDS treatment and care. According to this socio-ecological model, HIV-related behaviour needs to be changed at every level: individual, interpersonal, community, institutional/health system, and structural [22]. Bangladeshi policymakers can consider incorporating this socio-ecological model to strengthen the national HIV/AIDS prevention and reduction program.

### Study strength and limitations

One of the strengths of this study is that we conducted a qualitative study instead of employing a quantitative research method like a survey using a structured questionnaire which might allow to collect information from a large number of respondents and generalize the study findings; but might not help to gather the relevant subjective information like exploring the experience, feelings, attitude, and perception of the study respondents. This justification was also supported by Austin & Sutton (2014) [99]. Moreover, we conducted an in-depth interview, during which the interviewers got the opportunity to create a good rapport with the study respondents before and during the interview [100]. Similarly, our interviewer got the chance to overcome the challenges of exploring even sensitive or complex topics like HIV infection and could collect more prosperous and precious data through her descriptive and narrative capabilities.

Furthermore, during the data collection period, the interviewer did not face any challenges, and the in-depth interviews were conducted smoothly. This happened mainly due to several reasons: formal approval of AAS’ Executive Director, the introduction of the research team to senior officials and counsellors of AAS by the Executive Director, use of a private room next to the senior counsellors’ room in the AAS’s premises for data collection, availability of counselling service for the respondents if they feel any distress, a detailed explanatory statement in local language which provides all sorts of the required information related to study including voluntarism and presence of no or minimal risk of joining the study.

Though we collected significant useful study findings, this study is not free from limitations. We anticipated that particular meanings could be lost while translating the raw study findings from Bangla to English. This limitation was overcome by the principal investigator and co-investigators, who are Bangladeshi and fluent in English and Bangla. They checked the transcribed and translated data collected by the research assistant before analysis. Thus, they ensured the quality of the collected data. We collected data from the study respondents receiving ART for at least one year to identify the barriers to treatment adherence. As a result, we could not collect data from the new PLHIV.

We also could not collect data from those PLHIV who might face the most substantial barriers and as a result, discontinued ART. This might happen because either they did not visit the AAS during the data collection period or were not interested in joining the study. It was not possible to identify those PLHIV who discontinued ART living in the community; hence, the research team could not make any attempt to invite or recruit them in the study. Moreover, the research team did not recruit the study respondents unless they voluntarily expressed their interest after knowing about the study from the advertisement.

Another study limitation is that our study respondents were predominantly male; therefore, we could not get a comprehensive perspective from the female study population’ point of view compared to the male study population. Furthermore, there were 20 transgender PLHIV, but no one expressed their willingness to join the study. So, we also could not collect their opinions.

We collected data from the Dhaka centre of the "Ashar Alo Society" (AAS), which is located in the capital city of Bangladesh, i.e., we could not collect data from Sylhet and Chittagong, northern and southern border districts of Bangladesh close to India and Myanmar borders where PLHIV were also highly concentrated. Therefore, we could not explore the barriers to treatment adherence faced by the PLHIV living in remote districts. This mainly happened because the research team did not get any funds to conduct this study; hence they could not make any arrangements to travel over there. Furthermore, we collected self-reported data on adherence without checking their medical history record and using any objective measures.

## Conclusion

Our study explored three significant categories of barriers at the individual, community and institutional levels that negatively interfered with ART adherence. The most dominant barriers were discrimination and rejection related to stigma at the community and institutional levels. These stigmatizing attitudes and the discriminatory act critically affect PLHIV’s ART adherence. The Bangladesh government is committed to ending the HIV/AIDS epidemic through comprehensive policy and interventions. We recommended strengthening Bangladesh’s HIV/AIDS prevention, treatment and management program by evaluating the program effectiveness, with special focus on improvement of the supply chain of ART-related medicines and CD4 tests to slow the HIV transmission. In addition, we recommended strengthening the behaviour change communication and IEC activities at large scale to destigmatize health facilities and community level. This may help to stop HIV/AIDS in Bangladesh by 2030.

## Supporting information

S1 Table Semi-structured guideline to conduct the in-depth interview. (DOCX)

## Abbreviations

AAS: Ashar Alo Society
AIDS: acquired immunodeficiency syndrome
ART: antiretroviral therapy
BCC: behaviour change communication
BSMMU: Bangabandhu Sheikh Mujib Medical University
CMH: Combined Military Hospital
IEC: Information Education Communication
HCP: healthcare providers
HIV: human immunodeficiency virus
IDH: Infectious Disease Hospital
MUHREC: Monash University Human Research Ethics Committee
PLHIV: people living with HIV
RA: research assistant
