# Supplementary material for: Exploring the barriers to the antiretroviral therapy adherence among people living with HIV in Bangladesh: A qualitative approach
Source: PLoS One. 2022 Oct 21;17(10):e0276575. doi: 10.1371/journal.pone.0276575 (PMC9586390; doi:10.1371/journal.pone.0276575)
Supplement: S1 Table — (DOCX) [file pone.0276575.s001.docx]

| **Semi-structured guideline to conduct the in-depth interview**  Study ID number:  Date of data collection:  **A. Socio-demographic Information:**  a) Age (in a completed year on January 01, 2019)  b) Sex  c) Highest education level  d) Employment status  **B. Medical history:**  a) When have you been diagnosed as a people living with HIV (PLHIV)?  b) Did you disclose your PLHIV status to anyone? If yes, who are they? If not, what is the reason behind it?  c) When did you start the antiretroviral therapy (ART)?  d) How would you describe your health since you started the ART?  **C. To assess the ART adherence:**  a) Over the last two days, can you tell us whether you have taken each medicine on time? If not, why did you not take it?  b) Have you ever missed any dose or an appointment at your ART clinic? If yes, can you please tell us why this happened?  c) Have you ever thought about stopping ART? If yes, please let us know why you think in this way?  **D. To explore the barriers to ART adherence:**  a) Have you had any experience of being treated differently because of your PLHIV status, which affects you to continue your ART? If yes, will you please share your experience in the context of family, workplace, community, and healthcare facility?  b) Did you face any barriers to continuing your ART? If yes, what are those barriers? What do you perceive as the most significant barrier to continuing your ART?  c) Please let us know how you overcome those barriers, if any?  d) Please also let us know your opinion on what could be done to help PLHIV to adhere to their ART. |
| --- |
